# Urinary biomarker N-acetyl-β-D-glucosaminidase can predict severity of renal damage in diabetic nephropathy

**DOI:** 10.1186/s40200-015-0133-6

**Published:** 2015-02-12

**Authors:** Gehan Sheira, Nashwa Noreldin, Almokadem Tamer, Mohamed Saad

**Affiliations:** Department of Internal Medicine, College of Medicine, University of Tanta, Tanta, Egypt; Clinical Pathology, College of Medicine, University of Tanta, Tanta, Egypt

**Keywords:** NAG, Diabetic nephropathy, Albuminurea

## Abstract

**Background:**

Diabetic nephropathy is a clinical diagnosis where proteinuria is present in a patient with diabetes. Early intervention can significantly improve the prognosis. However, imprecision of the currently available biomarkers have impaired effective therapies in a timely manner. Urinary N-acetyl-β-D-glucosaminidase (NAG) is excreted in abnormally high amounts in many renal diseases. The aim of this study was to evaluate urinary NAG as an early biomarker in detection of diabetic nephropathy and whether it parallels the severity of kidney damage in different stages of diabetic nephropathy.

**Methods:**

Fifty patients with type 2 DM were classified into 3 groups (normoalbuminurea, microalbuminurea and macroalbuminurea) and 10 healthy subjects served as a control group. Urinary NAG, albumin and creatinine were measured. Blood urea, serum creatinine, serum albumin, total proteins, serum cholesterol, alanine aminotransferase (ALT), aspartate aminotransferase (AST), fasting and postprandial blood glucose, HbA1c and creatinine clearance were measured for all subjects.

**Results:**

All diabetic patients had a significantly higher level of urinary NAG compared to control. NAG value increased in parallel with the severity of renal involvement.

**Conclusion:**

Urinary NAG expresses the degree of renal impairment in diabetic nephropathy.

## Background

Diabetic nephropathy occurs in 20–40% of patients with diabetes and is the leading cause of chronic kidney disease and end-stage renal disease [1,2]. The onset of elevated levels of urinary albumin excretion is an early sign of diabetic nephropathy. It has been shown in diabetic patients that, microalbuminuria predicts the occurrence of macroalbuminuria and renal function decline [3]. As a result, high albuminuria has become an established renal risk marker in these patients [4]. However, urinary albumin excretion can be affected by several factors including plasma concentrations of atrial natriuretic peptide, arginine vasopressin, angiotensin II, aldosterone and fasting blood glucose, glycated hemoglobin, and mean arterial blood pressure [5] and albumin can be degraded in a manner consistent with the activity of endogenous urinary proteases. Furthermore, the inter-individual variation is as high as 47%. Because of these problems, all results that are initially positive for albuminuria need to be confirmed with a second sample collected on a different day, and in cases of discrepancies between the first and second sample, a third sample is necessary [6].

Several tubular damage markers recently have been discovered. Increased levels of these markers are supposed to indicate proximal tubular damage in the case of kidney injury molecule (KIM)-1, neutrophil gelatinase–associated lipocalin (NGAL), N-acetyl-β-D glucosaminidase (NAG), and cystatin C and distal tubular damage in the case of heart fatty acid–binding protein (H-FABP). These tubular damage markers have been extensively investigated in the field of predicting the occurrence of acute kidney injury after various nephrotoxic insults, such as ischemia during cardiac surgery, sepsis, and administration of contrast medium [7,8]. Little research has been done in patients with chronic kidney disease. In this study, we aimed to investigate urinary level of the N-acetyl-β-Dglucosaminidase (NAG) as proximal tubular damage marker in diabetic patients and non-diabetic control subjects in order to evaluate the relation of this marker to the severity of kidney disease as assessed by albuminuria and the estimated glomerular filtration rate (eGFR).

## Methods

This study was conducted in the Internal Medicine Department, Tanta University Hospital from April 2013 to October 2013. After approval of the Institutional Review Board of the College of Medicine, Tanta University, Egypt, fifty type 2 diabetic patients with initial diagnosis of diabetes at >30 years of age were recruited into the study. They were collected from inpatients and also from outpatients who were followed in the diabetic clinic. Hospitalized diabetic patients enrolled in our study were non-critically ill and their samples were not collected until they had fully recovered from their medical illness. Patients were stratified by the extent of albuminuria in the first morning urine void. Besides, we included 10 non diabetic volunteers as controls. Patients were divided into 4 groups: Group I: 10 non diabetic non hypertensive healthy persons as a control group, group II: 10 patients with normoalbuminuria, albumin creatinine ratio (ACR) ≤3 mg/mmol), group III: 20 had microalbuminuria ACR 3–30 mg/mmol, group IV: 20 have macroalbuminuria ACR >30 mg/mmol.

Written informed consent was obtained from all participants and the privacy of the data was considered. Exclusion criteria included patients with liver diseases, cardiovascular diseases, cancer, infections or inflammatory conditions, renal disease other than diabetic nephropathy and pregnancy. Patients and volunteers were fasted for at least 6 hours and were asked to abstain from unaccustomed physical activity for the preceding 24 hours. All subjects rested for 30 minutes in a supine position prior to initial blood sampling. All patients included in the study were subjected to full history taking, complete clinical examination (including blood pressure, fundus examination), ECG for exclusion of cardiovascular disease, laboratory investigations included: glucose, HbA1c, renal function tests, glomerular filtration rate by Cockcroft-Gault Equation Creatinine clearance = { [140 - age (years)] × weight (kg)} / [72 × serum creatinine (mg/dl)] × (0.85 if female), serum albumin, total proteins, Alanine aminotransferase (ALT) and Aspartate aminotransferase (AST), cholesterol levels and urine analysis that included: albumin concentration, urine creatinine and urinary concentrations of NAG. The kit uses a double antibody sandwich enzyme Linked immunosorbent assay (ELISA). Catalog No. MB 5700742.

Statistical analysis of the data of the present study was conducted with SPSS Version 16 using the mean, standard deviation and Yates’ corrected chi-square test. One-way ANOVA was used to compare means from all groups. The relationship between urinary NAG and other parameters was determined using the Spearman correlation analysis and the linear regression method. P values 0.05% were considered to be statistically significant.

## Results

Fifty diabetic patients were divided into three groups according to albumiurea and 10 control subjects were included in our study. No patient was excluded for any reason. Demographic data were comparable between groups while duration of the disease was significantly longer in groups III and IV compared to group II (Table 1). A total of 13 inpatients were enrolled in our study; 3 in group II and 5 in each of group III and VI. They were admitted because of uncontrolled hyperglycemia (7 patients), chest infection (6 patients).

**Table 1:** **Demographic characteristics**

**Variable**	**Group I**	**Group II**	**Group III**	**Group VI**	**P**
Age	47.30 ± 4.63	51.36 ± 5.25	48.64 ± 5.74	52.84 ± 7.63	0.951
Gender M/F	6/4	8/2	10/10	8/12	0.635
Disease duration	-	8.63 ± 3.25	13.2 ± 4.63	12.4 ± 2.34	0.035*

M = male, F = female, * = significant.

HbA1C, albumin/creatinine ratio, serum creatinine and serum urea showed statistically significant increase in group IV compared to group III and group III compared to group II and the all groups compared to group І (Table 2).

**Table 2:** **Laboratory and calculated data**

**Variable**	**Group I**	**Group II**	**Group III**	**Group VI**	**P**
HbA1C	5.10 ± 1.24	6.40 ± 2.42	7.86 ± 2.85	8.60 ± 3.17	0.006*
Albumin/creatinine ratio	1.53 ± 0.87	2.36 ± 0.95	21.69 ± 5.36	45.35 ± 13.25	0.001*
Serum creatinine mg/dl	0.75 ± 0.11	0.86 ± 0.13	1.60 ± 0.23	2.08 ± 0.63	0.008*
Serum urea mg/dl	32.14 ± 2.24	37.85 ± 5.64	55.96 ± 9.31	66.24 ± 10.24	0.002*
Estimated GFR ml/min	110 ± 25.36	93 ± 19.52	70 ± 15.49	56 ± 12.69	0.001*
Serum cholesterol mg/dl	180.6 ± 32.25	201.6 ± 29.60	214.9 ± 25.43	231.5 ± 29.6	0.020*
Urinary NAG ng/ml	0.93 ± 0.15	1.15 ± 0.25	1.33 ± 0.36	1.59 ± 0.42	0.001*

* = significant.

Estimated GFR, serum cholesterol, urinary NAG showed a statistical significant decrease in group IV compared to group III and in group III compared to group II and in the all groups compared to group І as showed (Table 2).

**Figure 1:**
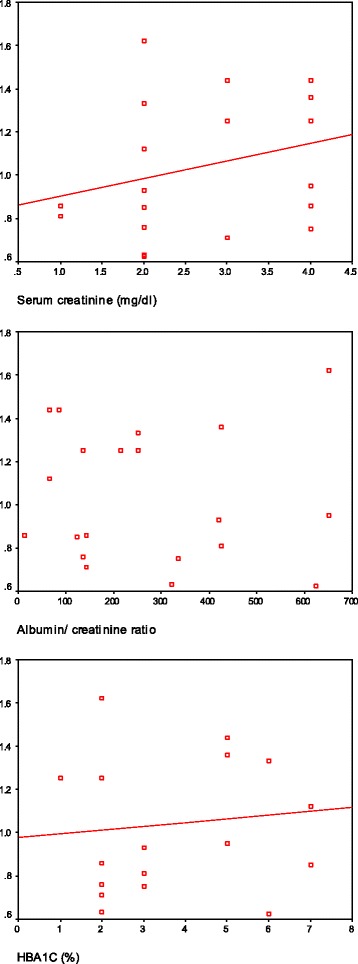
**Correlation between NAG and serum creatinine, albumin/creatinine ratio and HBA1C.**

Significant positive correlation was observed between urinary NAG and albumin/creatinine ratio (*P* = 0.006), serum creatinine (*P* = 0.002) and HBA1C (*P* = 0.008) (Figure 1).

## Discussion

Our results revealed that there was significant increase in HbA1c in (macroalbuminuric and microalbuminuric) diabetic patients than (normoalbumiuric diabetic patients and healthy controls) and this coincides with other studies [9-13] that they recorded that Poor glycemic control is a well-known risk factor for most diabetic complications, not only diabetic nephropathy.

Comparison between all the studied groups as regard Albumin/creatinine ratio, serum creatinine and serum urea showed statistically significant increase in group IV than group III and group III than group II and the all groups than group І. This is agreement with the findings of Mitsnefes et al. [14] who reported that higher levels of blood urea and creatinine indicate a failing of GFR as a result of decreased capability of the kidney to excrete waste products. However, Devarajan [15] in chronic kidney disease, found that blood urea will only be elevated above the normal when more than 60% of kidney cells are no longer functioning and the usefulness of blood urea as an independent indicator or renal function is limited the variability of its blood levels as a result of non-renal factors such as gastro-intestinal hemorrhage, mild dehydration, high protein diet and decrease perfusion of the kidneys.

Our study revealed that eGFR was significantly lower in diabetic patients than that of the healthy control group, Similar findings were previously recorded by others [16-20]. Moreover Jamal et al. [21] in their retrospective study to assess the factors affecting the progression of diabetic nephropathy and its complications, they showed an average of 3.3 mL/year drop in GFR and even greater among patients who reached ESRD (5.9 mL/year) and the presence of persistent proteinuria was a strong risk factor for subsequent loss of GFR so they re-emphasizing earlier reports that established the importance of sustained increases in urine albumin excretion in the pathogenesis and diagnosis of diabetic kidney disease.

In this study, diabetic nephropathy was associated with elevated urinary NAG values compared to a control group. This increase in NAG was parallel to the severity of renal involvement with a characteristic increasing trend was observed among the three groups regarding albuminuria. These results are in agreement with other studies [22,23] that stated that changes in urinary NAG activity can reflect the activity of the disease as well as the residual functional capacity of the kidney. Our results showed that even in the absence of any clinical evidence of microvascular complications, urinary NAG excretion was invariably elevated indicating that subclinical renal tubular dysfunction may exist before the occurrence of glomerular damage. Kuzniar et al. [24] denoted that in proteinuric glomerular diseases the increased NAG excretion can occur even in absence of morphological evidence of tubular cell damage, probably reflecting increased lysosomal activity of these cells due to the increased uptake of filtered proteins. Also Navarro et al. [25] stated that NAG changes occur prior to microalbuminuria, probably because the tubular cells can reabsorb the increased albumin load that results from glomerular affection but the increased NAG will be lost from the damaged cells. Also, Nauta et al. [26] showed that urine NAG increases a surprising 9-fold in normoalbuminuric patients with diabetes compared to controls. It increases further with development and progress of microalbuminuria. Moreover, in another study of diabetic patients, Vaidya et al. [27] found, that urinary levels of tubular injury biomarkers KIM-1 and NAG were significantly elevated in patients with type 1 diabetes and macroalbuminuria in comparison with diabetics with normoalbuminuria and nondiabetic healthy controls. Low urinary KIM-1 and NAG at baseline estimation were strongly associated with regression of MA during a 2-year follow-up. It is generally agreed upon that there is some normally filtered albumin that is reabsorbed by the proximal tubule. If this reabsorption is impaired, we would expect to see macroalbuminuria; therefore, the earliest kidney lesion in type 1 DM may be tubular injury, not glomerular injury. Also urinary NAG elevation would be expected to be enhanced with proximal tubule injury although it may also be increased because of enhanced lysosomal activity without injury. Also Kern et al. [28] showed the change in NAG independently predicted macroalbuminuria. Thus, the risk for macroalbuminuria raised more than two fold for every 50% increase in urine NAG at baseline, and additionally rose almost 9% for every unit increase in urine NAG across the time interval from baseline to the occurrence of macroalbuminuria. Moreover another study [29] denoted that urinary NAG might be elevated in other diseases other than diabetic nephropathy. They investigated 136 patients with primary glomerulonephritis [74 with idiopathic membranous nephropathy (IMN), 44 with primary focal segmental glomerulosclerosis (FSGS) and 18 with minimal change disease (MCD)] they found Urinary NAG excretion can be considered as a reliable marker of the tubulo-toxicity of proteinuria in the early stage of IMN, FSGS and MCD; the excretion values show a significant relationship with 24-h proteinuria, Also Orfeas et al. [30] found that patients in the highest urinary NAG had a significantly higher prevalence of sepsis, higher Acute Physiology and Chronic Health Evaluation (APACHE) II score, Multiple Organ Failure (MOF) score, a higher likelihood of mechanical ventilation, higher prevalence of oliguria, and fractional excretion of sodium 1% as compared with those with lower urinary NAG. Also Dialysis requirement and hospital death were significantly higher in the high urinary NAG level patient as compared with the low urinary NAG level patient.

Correlation between Urinary NAG level and Albumin/creatinine ratio, serum creatinine and HbA1c showed significant + ve Correlation between Urinary NAG level and all previous parameters , this coincides with Sato et al. [31] stated that NAG total activity and A2 isoenzyme were highly correlated with the level of proteinuria with A2/A1 ratio higher in glomerular diseases (e.g. glomerulonephritis and diabetic nephropathy) than tubulointerstitial pathologies (e.g. chronic pyelonephritis and polycystic kidney). Also Abdelshakoer et al. [23] showed that Urinary NAG was strongly affected by the blood glucose control over the 1–7 days before urine collection.

Our study is not without limitations. The number of patients might be not enough to determine the reliability and generalizability of urinary NAG levels as expressing the degree of renal impairment in diabetic nephropathy. Larger studies are needed to confirm or refute our results. Also, our results remain limited to our patient population in the fact that other factors than proteinuria may play a role in the increased urinary NAG excretion in diabetics like blood glucose control [32].

## Conclusion

Based on the limitations above, it can be concluded that urinary NAG levels could be used as a useful biomarker reflecting the degree of renal impairment in diabetic nephropathy.
